# Natural Food Polysaccharides Ameliorate Inflammatory Bowel Disease and Its Mechanisms

**DOI:** 10.3390/foods10061288

**Published:** 2021-06-04

**Authors:** Yikun Wang, Haibin Zhu, Xiaoji Wang, Yue Yu, Jianhua Xie

**Affiliations:** 1 Queen Mary School, Jiangxi Medical College, Nanchang University, Nanchang 330006, China; yiqueenwang@163.com; 2 State Key Laboratory of Food Science and Technology, Nanchang University, Nanchang 330047, China; zhuhaibin163@163.com (H.Z.); zongyu_li@email.ncu.edu.cn (Y.Y.); 3 School of Chemical Engineering and Energy Technology, Dongguan University of Technology, Dongguan 523808, China; wangxj@dgut.edu.cn

**Keywords:** polysaccharides, inflammatory bowel disease, mechanism

## Abstract

Natural polysaccharides and their metabolites’ short chain fatty acids (SCFAs) have attracted much attention. Recently, they have shown great potential in attenuating systemic inflammation activities, especially in inflammatory bowel disease (IBD). IBD is a complex pathological process and is related to epithelial damage and microbiota imbalance in the gut. Recent studies have indicated that natural polysaccharides could improve IBD recovery by different mechanisms. They could not only influence the ratio of intestine microbiota, but also regulate the secretion levels of immunity cytokines through multiple pathways, the latter including modulation of the TLR/MAPK/NF-κB signaling pathways and stimulation of G-protein-coupled receptors. Moreover, they could increase intestinal integrity and modulate oxidative stress. In this review, recent research about how natural polysaccharides impact the pathogenesis of IBD are summarized to prove the association between polysaccharides and disease recovery, which might contribute to the secretion of inflammatory cytokines, improve intestine epithelial damage, reduce oxidative stress, sustain the balanced microenvironment of the intestines, and finally lower the risk of IBD.

## 1. Introduction

Inflammatory bowel disease (IBD) is represented by ulcerative colitis (UC) and Crohn’s disease (CD), which manifest as chronic inflammation of the gut [1]. It has a higher prevalence and a rising incidence in developing countries in recent years [2]. The clinical features of IBD include pathological damage, ulceration of intestinal mucosa, diarrhea, as well as bloody stool [3]. Various factors have been involved in the pathogenesis of IBD, such as environmental exposure, diet shortage, innate and required immunity deficiency, or gut microbiota dysregulation [4,5]. Until now, the conventional treatment of IBD has still used drugs such as methylprednisolone, infliximab, and cyclosporine. However, the continued refractory as well as the serious adverse effects are strongly troubling to the patients [6]. Recently, it has been indicated that polysaccharides from the natural source could alleviate IBD symptoms, which might be an alternative element in the treatment of IBD [7].

Natural polysaccharides can improve intestine damage recovery, through their structural characteristics, molecular weight, and bioactivities. The diversity of microbiota is significantly reduced comparing the IBD patient with normal people. The microbiota dysbiosis in the gut is recognized as the increase of *Proteobacteria* and *Bacteroidetes*, and simultaneously a decrease in the abundance of *Firmicutes*, *Lactobacillis* and *Clostridia* [8,9]. Polysaccharides can be metabolized by gut microbiota and finally fermented into SCFAs, then SCFAs modulate the gut microbiota ratio via increase the abundance of probiotics and decrease the ratio of bacteria harmful to the gut mucosa.

Recently, more and more research has attached great importance to the immunity effect in the IBD development [10]. On the one hand, the supplement of natural polysaccharides up-regulates the tight junction proteins, which not only decreases the neutrophil infiltration through intestine mucosa, but also promotes the recovery of the epithelial barrier of the colon. On the other hand, administrating polysaccharides can influence the production of inflammatory cytokines in the IBD patients’ gut. As the indispensable component of the immune responses, inflammatory cytokines send signals to the numerous immune cells, which are critical to cellular immunity regulation. Excessive pro-inflammatory cytokines, like interleukin (IL)-6, IL-1β, tumor necrosis factor (TNF)-α, induce diseases associated with immunity reaction, while anti-inflammatory cytokines ameliorate the inflammation and promote recovery [9]. Thus, recovering the balance of inflammatory cytokine production via natural polysaccharides is an essential mechanism to the gut defensive response.

The excretion of inflammatory cytokines is regulated by the inflammatory signaling pathways including toll-like receptors (TLR), mitogen-activated protein kinase (MAPK), nuclear factor-κB (NF-κB), and G-protein coupled receptors, which participate in regulating the phosphorylation level of TLR2/4/6, ERK, IκB, GPR41, and GPR43 [11]. By regulating these signal transduction pathways, natural polysaccharides and their metabolites ameliorate IBD symptoms in the damaged tissues. Polysaccharides alleviate oxidative stress through down-regulating the production level of oxygen free radicals including myeloperoxidase (MPO), nitric oxide (NO), malondialdehyde (MDA), which subsequently facilitate IBD symptoms [12].

Most of the reviews related to natural polysaccharides have paid attention to their structures and anti-inflammatory applications in pharmaceuticals. However, few discussed the effects of natural polysaccharides and their mechanism involved in colitis in detail and in its entirety. This current review summarizes the latest linkages of natural polysaccharides and IBD, including the types of natural polysaccharides that alleviate the IBD symptoms and the mechanisms of moderation of this disease. The main objective is to discuss the relationship between natural polysaccharides and IBD treatment.

## 2. Polysaccharides and Their Impact on Inflammatory Bowel Diseases

### 2.1. Polysaccharides from Hericium Erinaceus

A purified polysaccharide (EP-1) isolated from *Hericium Erinaceus* has been studied for its anti-gastric/colitis ulcer activity [11]. The molecular weight of EP-1 is around 3.1 kDa and the structure is constituted of glucose, mannose, and galactose, whose backbone is α-d-Glc(1→3) and β-d-Glc(1→3) and both of them are branches with C-4 position [13].

Recently, EP-1 has been confirmed to relieve the symptoms of ulcerative colitis induced by acetic acid [14]. As it enters the lower gastrointestinal tract, the polysaccharide would be fermented by the gut microbiota to form SCFAs. The carbon from different polysaccharides gets used as the unique energy source for particular microbes in intestine [15]. Therefore, the polysaccharides could change the composition of the gut microbiota, which is companied with changes of the SCFAs. EP-1 changes the bacteria community through recovering the levels of *Firmicutes, Bacteroidetes, Proteobacteria*, and *Actinobacteria* with the increase of SCFAs, including ethanoic acid and butanoic acid, in the colon. SCFAs including acetate, propionate, and butyrate are the last degraded product metabolized from polysaccharides by the gut microbiota [16], which influence the diversity of intestinal microbiota, and contribute vitally to immune response regulation in the gastrointestinal system [17].

Another polysaccharide was extracted from *Hericium erinaceus*, whose molecular weight is 86.67 kDa. It was also reported to down-regulate the expression of IL-6, IL-1β, TNF-α, and cyclooxygenase-2 (COX-2); induce iNOS; and decrease the expression of related mRNA in dextran sulfate sodium (DSS)-induced mice [18]. iNOS belongs to the systems of reactive oxygen/nitrogen species (ROS/RNS) generation that stimulate oxidative stress. Oxidative stress is tightly connected with the production of inflammatory cytokines and inflammatory cell penetration [19]. As the polysaccharides reduce the expression of iNOS, there is the amelioration of oxidative stress. Otherwise, iNOS stimulates the manufacture of the NO and promotes the expression of COX-2; the latter could catalyze the prostaglandin production finally induced the inflammation responses. All of these implementations depend upon the NF-κB signaling pathway [20].

### 2.2. Sarcodon aspratus Polysaccharides

The water-soluble polysaccharide (HCP) extracted from *Sarcodon aspratus* with a molecular weight of 6.7 × 10^3^ kDa was reported having immunomodulatory function in the intestine. HCP has a backbone structure of (1→6)-linked-α-d-glucopyranosyl residues. HCP facilitates the immunity function of macrophages cells through the TLR4-MAPK-NFκB signaling pathway, which enhances the phagocytic activity and promotes the expression of NO, iNOS. HCP works toward regulating the immunity in the intestine through moderating inflammatory cytokines secreted from immunity cells [21].

### 2.3. Dictyophora indusiata Polysaccharide

*Dictyophora indusiata* polysaccharide (DIP) is isolated from mushrooms with a molecular weight of 1132 kDa. The main linkage-type of DIP including (1→3)-linked α-L-Man, (1→2, 6)-linked α-d-Glc, (1→6)-linked β-d-Glc, (1→6)-linked β-d-Gal, and (1→6)-linked β-d-Man. DIP could markedly facilitate the excretion of NO, TNF-α, and IL-6 from macrophages involved in complement receptor 3 (CR3) [22]. DIP has a latent treatment impact against colitis by increasing the level of *Bacteroidaceae* and *Enterobacteriaceae* in the colitis microenvironment. The administration of DSS-induced mice with DIP significantly reduced the pro-inflammatory cytokines (TNF-α, IL-1β, IL-6, IFN-γ) and enhanced anti-inflammatory cytokines (IL-4, IL-10) [23]. The treatment of DIP with the DSS-induced mice restrains the biomarker of M1 macrophages CD86. It changed the macrophage subset distribution by reducing the production of M1 subsets from splenic macrophages and made deviate polarization to M2 subsets [24]. The macrophages have a tight connection to the inflammatory cytokines’ generation. The M1 macrophages are connected to IL-6 as well as TNF-α, and M2 macrophages are associated with the generation of IL-10 [25]. DIP also can down-regulate the MPO level dramatically in a dose-dependent manner. As an inflammatory marker, the association between MPO and IBD is that it identifies the infiltration of inflammatory cells and tissue damage. Simultaneously, DIP also decreases NO activity and increases T-SOD levels. The latter would give rise to the production of NO [26], which triggers tissue damage and induces inflammatory responses during the occurring of IBD [18].

Recent studies have found that DIP not only decreased endotoxins, including lipopolysaccharides, but also maintained the normal level of tight junction (TJ) proteins expression including claudin-1, occludin, and zonula occludens (ZO)-1 in the antibiotic-induced mice [27]. Broad spectrum antibiotics have been demonstrated to cause gut dysbiosis [28] and destroy epithelial barrier integrity [29], thus raising the hazard of IBD occurrence in the neonates [30]. DIP could restore the function of the gut barrier. The gut mucosal barrier prevents the pathogens and toxins from invasion into the intestine, which has an essential function involved in maintaining the intestine in healthy condition. Additionally, DIP also inhibited the colonic tissue apoptosis in the ulcerative colitis mice by improving the expression of Bcl-2 and Bax [24].

### 2.4. Flammuliana velutipes Polysaccharids

Flammuliana velutipes polysaccharides (FVP) are comprised of glucans, galactan, xyloglucan, and xylomannan, whose backbone is α-(1→4)-d-glucose residues with branches consisting of (1→6)-linked α-d-glucopyranosyl [31]. FVP has various bioactivities including immunity regulation [32], anti-oxidant, anti-microbial, anti-tumor properties, and so on [33]. FVP could recover colon injury induced by DSS, and moderate the dysbiosis in the gut microbiota, such as by boosting the *Firmicutes/Bacteroidetes* ratio and enhancing the *Lachnospiraceae* abundance [34]. *Lachnospiraceae* is able to restrain intestinal immune homeostasis via promoting the SCFA production [35], which might explain the phenomenon that FVP increased the production of SCFAs in the gut.

### 2.5. Pleurotus eryngii Polysaccharides

Treatment of *Pleurotus eryngii* polysaccharides (PEP) could increase the abundance of *Porphyromonadaceae, Rikenellaceae, Bacteroidaceae,* and *Lactobacillaceae* on C57BL/6 mice. More importantly, PEP increased the SCFA production in the mice colon. SCFAs exert numerous functions in the mice including regulating gut immunity conditions, changing the mucus layer composition and restraining the homeostasis of the intestine [36]. At the same time, PEP inhibited the expression of ROS and NO in RAW264.7 macrophages, and sulphonated PEP showed more powerful anti-inflammatory activity [37].

A water-soluble polysaccharide (EPA-1) isolated from *Pleurotus eryngii* (99.7 kDa) consisting of mannose, glucose, and galactose with the ratio of 2.2:1.0:3.2 EPA-1 not only increased the manufacturing of NO and pro-inflammatory cytokines including TNF-α, IL-1, as well as IL-6, but also exhibited the improved expression level of phosphorylated p38, ERK, JNK, and NF-κB. It reflects the EPA-1 influence inflammatory response via the effect on MAPK and the NF-κB signal mediated pathway [38]. EPA-1 exerts intestine immunity to enhance its protective effect. The immunity cytokines including IL-6 motivated signaling pathways could promote the various leukocytes recruiting and activation [39], which is subsequently followed by possessing invading microorganisms and further diminishment of epithelial damage. Therefore, EPA-1 ameliorates IBD through moderate inflammatory cytokines. Above all these results have indicated that polysaccharides from *Pleurotus eryngii* have the function to alleviate IBD. Therefore, it is thought that PEP reduces the risk of IBD and benefits colitis recovery [40].

### 2.6. Gracilaria Polysaccharides

*Gracilaria caudata* polysaccharide consists of 1→3-linked-β-d-galactose and 1→4-linked-3,6-anhydro-α-L-galactose [41] with a molecular weight of 116 kDa [42]. Administrated *G. caudata* polysaccharide could significantly reduce neutrophil infiltration in acetic acid-induced mice via decreasing the expression of MPO levels together with restoring the colon from acetic acid injuries by reducing oxidative stress, including the diminishment of GSH consumption and iNOS expression in the gut [43].

Another species from genus of *Gracilaria* is *Graciliaria lemaneiformis*, the polysaccharides of *Graciliaria lemaneiformis* (SP), which also has sulfated groups. In the mice model developed by Ren et al. [18], SP was orally administrated 200, 400, 600 mg/kg/day for 3 weeks and the arresting weight loss following subsequently by the improved conditions of mice. Histological analysis showed that the colon structure, including the crypt number and muscular structure, was improved by raising the SP dosage, suggesting that a high dose of SP protects the goblet cells and crypt structure in the gut. SP also could repress the intestinal endotoxin and lipopolysaccharide-binding protein production, together with the reduction of TNF-α, IL-6, and IL-1β expression [44]. All of these markers indicate that SP alleviates the symptoms of IBD.

### 2.7. Blidingia minima Polysaccharides

*Blidingia minima* often grow in Subei Shoal in China and have similar morphology characteristics as *Enteromorpha* species. Polysaccharides extracted from *Enteromorpha* species have revealed various beneficial functions such as, for instance, anti-inflammatory and anti-oxidant activities [45]. Recently, Song et al. administrated *Blidingia minima* polysaccharides (BMP) to DSS-induced mice, and found a decrease in the expression of anti-inflammatory cytokine IL-10 and an increase in the production of pro-inflammatory cytokines including IL-1β and TNF-α. The expression of NF-κB, IκB-α, and AKT was also increased, thus, BMP might modulate the motivation of NF-κB and AKT signaling pathway to ameliorate IBD [46].

### 2.8. Arctium lappa L. Polysaccharides

*Arctium lappa* L. is one of the well-studied traditional medicines. The polysaccharide (ALP-1) isolated from *Arctium lappa* L. has the molecular weight of 5.12 kDa and is constituted of a (2→1)-β-d-fructofuranose backbone linked to a terminal of (2→1)-α-d-glucopyranose at the non-reducing end and a (2→6)-β-d-fructofuranose branching. The anti-inflammatory impression of this alkaline extraction polysaccharide is thought to relate to its specific structure feature the (2→1)-linked type in fructan [47]. ALP-1 caused a higher relative abundance of Lactobacillaceae, Lachnospiraceae, as well as Ruminococcaceae, and a lower relative abundance of Bacteroides plus *Staphylococcus* in DSS-induced male ICR mice. It also led to a remarkable decline of pro-inflammatory cytokines such as IL-1β, IL-6, TNF-α, and improved levels of anti-inflammatory cytokines including IL-10 and immunoglobulin A (Ig A) in DSS-induced male ICR mice [48]. The pathogenic microorganisms are easier to be phagocytosed through phagocytic cells [49]. Similar effects were noted in another study conducted by Zhang and his coworkers where the same inflammatory cytokine results were observed when treating with ALP-1 in lipopolysaccharide (LPS)-induced RAW264.7 macrophage [50].

An alkali-soluble polysaccharide (ASPP) has proved to have a stronger anti-inflammatory and immunomodulatory effect [47,51]. Female ICR mice were administrated with ASPP 400 mg/kg body weight for 28 days. They displayed a higher abundance of probiotics such as *Firmicutes, Alistipes, Odoribacter, Lachnospiraceae,* as well as *Lactobacillus* in mice [52]. *Lactobacillus* not only has a reverse relationship with IL-1β but also prevents epithelial barrier disruptions by blocking TNF-α [53]. There is also an increment in the *Firmicutes/Bacteroides* ratio, which has a positive connection with a protective effect on IBD patients [54]. Except for elevating this ratio in the colon, ASPP simultaneously decreases the level of the harmful bacteria *Proteobacteria,* which boosts the excessive pro-inflammatory cytokines expression [55]. Otherwise, ASPP inhibits the expression of pro-inflammatory cytokines including IL-6, IL-1β, TNF-α, and IL-10 and decreases the overproduction of NO through down-regulating iNOS expression [56]. As one of the pro-inflammatory molecules, NO can stimulate the immune system [57] and is mediated by NF-κB and MAPKs pathways [58].

### 2.9. Morinda citrifolia L. Polysaccharides

*Morinda citrifolia* (Noni) has been utilized as medicine for a hundred years. Polysaccharides extracted from noni fruit (NFP) have been shown to exert numerous benefits such as being anti-diabetic, anti-inflammatory, anti-oxidant, and anti-cancer [59]. NFP is comprised of galacturonic acid, rhamnogalacturonan-I, arabinogalactan, and arabinan with a molecular weight of nearly 456 kDa. NFP could improve the tight junction protein production, including ZO-1 and occludins, and thus enhance the gut barrier integrity and decrease the possibility of endotoxemia in DSS-induced IBD mice [60].

### 2.10. Astragalus membranaceus Polysaccharides

*Astragalus membranaceus* has been used for curing numerous conditions such as wounds, diabetes, leukemia, eye disease, and nephritis for several centuries [61]. Recent studies have proved that the *Astragalus* polysaccharides (APS) could exert immunomodulation and anti-oxidant effects. In DSS-induced male C57BL/6 mice, APS was administrated 200 mg/kg/day for the first 3 days. It promoted the colitis-related clinical indices, and noticeably, when compared with the DSS-induced colitis group, a histopathology assessment revealed that all of the ulcerations and mucosal, edema, and neutrophil infiltration in the epithelium of intestine were significantly reduced. APS also down-regulated production of MPO and pro-inflammatory cytokines including TNF-α, IL-1β, and IL-6 as well as the expression of NF-κB in DSS-induced mice. Therefore, APS could relieve symptoms of colitis through modulating the NF-κB signaling pathway [62].

### 2.11. Dendrobium officinale Polysaccharides

*Dendrobium officinale* has been used as a Chinese medicine for thousands of years. Previous studies have displayed that the polysaccharides from *Dendrobium officinale* (DOP) make a significant contribution against inflammation and immunity modulating [63]. Recently, based on the specific endo-β-1,4-mannanase linear structure of DOP, Zhang et al. have separated the core domain of Dendrobium officinale polysaccharides (EDOP) after enzymatic. It consists of glucose and mannose with the molar ratio of 1.00:4.76 and is composed of (1→4)-β-d-Glcp and (1→4)-β-d-Manp with attached 2-O-acetylated groups [64]. The effects of EDOP on colitis were evaluated in DSS-induced colitis male Balb/c mice. Both DOP and EDOP could reduce the overexpression of pro-inflammatory cytokines including IL-1β, IL-6, and TNF-α together with their mRNA expression levels simultaneously, which could moderate the IBD to some extent. DOP and EDOP treatment increased the abundance of *Bacteroides, Lactobacillus,* plus *Ruminococcaceae*, and down-regulated the level of *Proteobacteria* and *Akkermansia* in the meantime. *Ruminococcaceae* is one of the common probiotics that could promote the degradation of complex polysaccharides followed by the increased production of SCFAs [65]. *Akkermansia* improves pro-inflammatory cytokine manufacturing and diminishes the SCFAs expression, as a result of a positive relationship with IBD [66]. DOP and EDOP also restored the mRNA expression level of GPR41/43, which reactivated the metabolism of SCFAs in the gut. These studies might explain that DOP and EDOP treatment recovered the level of acetic acid in the colon, thus promoting SCFA production in the gut. DOP can ameliorate IBD through exhibiting depression of NLRP3 inflammasome and β-arrestin1, following by subsequently regulating the expression of TLR-2,4,6,9 [67].

### 2.12. Lycium barbarum Polysaccharides

*Lycium barbarum* (Goji berry) has been utilized in medicine and diet for thousands of years. The major backbone of the polysaccharide isolated from *Lycium barbarum* (LBP) is comprised of (1→3)-β-d-galactopyranosyl, (1→6)-β-d-galactopyranosyl, and (1→4)-α-d-galactopyranosyluronic acid residues, and the molecular weight cover from 2.1-6.5 × 10^3^ kDa [68]. The supplementation of LBP promoted growth of intestinal probiotics such as *Akkermansia, Lactobacillus,* and Prevotellaceae. It also significantly increased the expression level of TGF-β and IL-6 in serum and sIgA in colon tissues [69]. Furthermore, treatment with LBP ameliorated the colitis symptoms in DSS-induced colitis C57BL/6 mice, which includes alleviating the damage of mucus and reducing the neutrophil infiltration in colonic tissues [68]. LBP could inhibit the production of chemokine ligand 1 (CXCL1), and monocyte chemoattractant protein 1 (MCP-1), together with the down-regulation of COX-2 [70]. CXCL1 is one of the potential chemo-attractants that could trigger neutrophils recruitment and activation in inflammation [71]. MCP-1 can attract immunity cells including monocytes, macrophages, and lymphocytes [72]. Both of them play an essential role in the occurring of colitis. The results have suggested that LBP might alleviate the IBD symptoms by restoring the normal level in the gut and decreasing the neutrophil infiltration.

### 2.13. Codonopsis pilosula Polysaccharides

*Codonopssi pilosula* polysaccharides (CREP) were used as an effective medicine for ulcerative colitis. CREP is comprised of mannose, rhamnose, galacturonic acid, glucose, galactose, and arabinose at the molar ratio of 1.00:3.26:27.87:10.87:7.70:9.94. Tang et al. found that CREP could ameliorate colitis symptoms in the DSS-induced mice combined with APS. In addition to the administrative effect, APS decreases the expression of pro-inflammatory cytokines and supplies CREP in the treatment, causing down-regulation of TJs proteins including occludin, ZO-1, claudins, and MUC-2 [73].

Especially, administration of CREP in the DSS-induced mice leads to the up-regulation of IL-22. IL-22 is one of the cytokines that belongs to the IL-10 family and can protect the gastrointestinal tract in the colitis models. A previous study has confirmed that the blocking IL-22 pathway prolongs recovery in the DSS-induced colitis model. IL-22 makes a crucial protective contribution to intestine integrity. On the one hand, IL-22 could facilitate the goblet cells’ secret mucins, which form the stabilized external barrier to sequester the direct interaction between epithelial cells and microorganisms [74]. On the other hand, it also could promote trauma healing through activating the STAT3 pathway in epithelial cells [75]. Therefore, CREP have potential effects to ameliorate IBD symptoms.

### 2.14. The Polysaccharides from Purple Sweet Potato

Purple sweet potato contains three polysaccharides consisting of water-soluble polysaccharide (WPSPP), dilute alkali-soluble polysaccharide, and concentrated alkali-soluble polysaccharide [76]. The backbone of the alkali-soluble polysaccharide (ASPP) obtained from purple sweet potato is composed of 1,4-linked Glcp with side chains attached to the O-6 position [77]. Sun et al. found that female ICR mice had higher levels of acetate and propionate when administrated with ASPP 400 mg/kg body-weight once a day for 30 days. ASPP inhibited the pro-inflammatory cytokines IL-1β, IL-6, and TNF-α, and the anti-inflammatory property of ASPP was by up-regulating the level of SCFAs [78].

A purified polysaccharide (WPSPP-1) from purple sweet potato was constituted of 1,6-α-d-Glcp, 1,4-α-d-Glcp, 1,2-α-d-Manp, and 1,4,6-α-d-Glcp with β-d-Glcp on the branches, and exhibited a molecular weight of 10^3^ kDa [79]. The administration of WPSPP-1 can promote SCFA production including acetate, propionate, and butyrate. It recovered inflammatory lesions through improving IL-10, SOD, and T-AOC levels and reducing IL-1β, TNF-α, IL-6 together with MDA levels. Meanwhile, it increased the ratio of Firmicutes/Bacteroides and decreased the proportion of Proteobacteria, exerting a beneficial effect on the colonic microbiota modulation [54].

### 2.15. Others

Polysaccharides (TFPS) from the flowers of *Camellia sinensis* L, which are comprised of two kinds of polysaccharides with a molecular weight of 4.4 kDa and 31 kDa [80] exhibit immunostimulant activity. TFPS have a probiotic-promoting effect which works through increasing the ratio of probiotics in IBD, which significantly changes the composition of the metabolite SCFAs. In IBD feces, the relative abundances of *Escherichia/Shigella, Enterococcus, Collinsella, Lactobacillus,* and *Bifidobacterium* were increased, while the relative abundances of *Enterobacter, Ptococcus, Bacteroides, Clostridium XlVa, Megasphaera, Roseburia, Granulicatella, Akkermansia* and *Fusobacterium* decreased [81].

The polysaccharides from *Ficus carica* (FCPS) were considered to be used in nutritional therapy due to their anti-oxidant and anti-inflammatory function. FCPS has a molecular weight of 98.9 kDa and is comprised of rhamnose, arabinose, glucose, galactose as well as galacturonic acid with ratios of 5.8:18.1:3.3:19.3:53.5. The administration of FCPS in DSS-treated mice could down-regulate the mRNA expression of IL-1β, TLR4, and iNOS; the high-dose FCPS essentially decreased the mRNA expression of TNF-α, IL-6, MCP1, and COX-2, which resulted in the inhibition of colon inflammatory response [82].

FCPS treatment could decrease the abundance of *Esherichia* and *Clostridium. Esherichia* always multiplies the danger of colitis and *Clostridium* is related to the production of SCFAs. The administration of FCPS can improve the level of *Prevotella, Bacteroides, Butyricicoccus*, and Coprococcus. All of them have been reported to be beneficial in preventing IBD through down-regulating the expression of SCFAs [83]. The effects of some natural polysaccharides on inflammatory bowel disease are shown in Table 1.

## 3. Mechanisms

**Figure 1:**
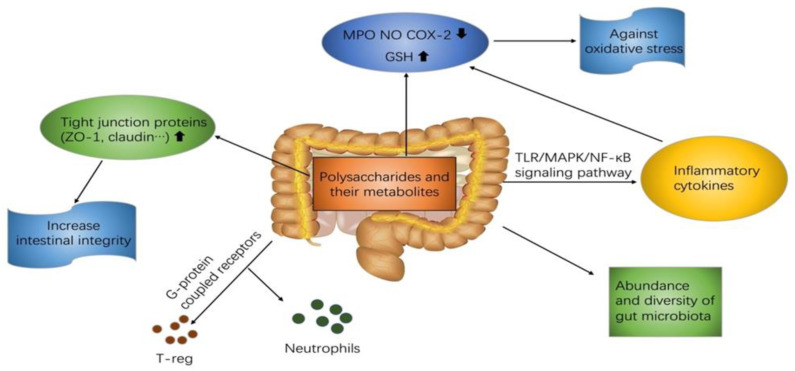
The effects of natural polysaccharides on IBD.

Natural polysaccharides regulate the expression of cytokines engaged in the inflammatory responses of IBD through multiple signaling pathways (Figure 1). Polysaccharides could ameliorate IBD symptoms via decreasing the neutrophil infiltration and regulation of oxidative stress. The following is a discussion of the potential mechanism of the effect of polysaccharides on therapy for IBD in Table 2.

### 3.1. TLRs-MAPK/NF-κB Mediated Signal Transduction Pathway

TLRs belong to the pattern recognition receptors and they are widely found on the external sides of immune cells such as macrophages, neutrophils, and lymphocytes. In the immunity signaling pathway, the process of TLR-recruiting adaptors enhances the production of numerous pro-inflammatory cytokines, which is followed by the activation of pro-inflammatory signaling pathways and host defense pathogens by participating in the signal transducer and activator [87]. After binding to polysaccharide ligands, TLRs motivate TNF receptor-associated factor 6 (TRAF6), which consequently activates MAPK and NF-κB. The anti-inflammatory impact of polysaccharide from *Sarcodon aspratus* was checked into LPS induced-RAW264.7 macrophage cells and it turned out to increase the production level of TLR4 as well as the activation of macrophage cells. It revealed that SAP exerts its protective effect on the intestine by enhancing the immunity reaction. Otherwise, when treating DSS-induced colitis mice with FCPS, it not only decreased the TLR4 expression but also down-regulated the manufacturing of pro-inflammatory cytokines including IL-1β, TNF-α, and IL-6 [82]. The outcome explains that the mechanism of FCPS alleviates IBD by inhibiting the immunity reaction.

MAPK is a family of serine/threonine protein kinases that are comprised of c-jun N-terminal kinase (JNK), p38, and extracellular signal-regulated kinase 1/2 (ERK 1/2) [88]. NF-κB is a transcription factor that is involved in the management of the expression of critical immunity and inflammation regulatory genes [89]. NF-κB always keeps an inactive complex form binding with inhibitory protein IκB in the normal condition [38]. As the cells are stimulated, NF-κB is activated and IκB is phosphorylated by IKK. Then, the activated complex form is transferred into the nucleus and regulates the expression of inflammatory cytokines and proteins. The underlying mechanism of polysaccharide- regulated intestine protective effects is considerably connected with the governed expression of MAPK and NF-κB. For example, when either of the polysaccharides obtained from *Ganoderma lucidum* and *G. sinense* or *Pleurotus eryngii* was administrated with LPS-induced RAW264.7 macrophage cells, the results revealed that all of the phosphorylation level of ERK, JNK, p38, and NF-κB were up-regulated [38,84], which indicated that all of these polysaccharides enhance the immune regulation effects. In another study, treating a high dose of DIP could significantly reduce the phosphorylation level of ERK and NF-κB and pro-inflammatory cytokines in DSS-induced mice [23]. These results are consistent with the previous results that polysaccharides have immunomodulatory activities on the immune cells, and further confirm that polysaccharides could ameliorate IBD via modulating the MAPK and NF-κB signaling pathway.

### 3.2. G-Protein-Coupled Receptors

G-protein-coupled receptors (GPRs) are a family of receptors for different second messengers. It has been demonstrated that GPRs highly expressed in the gastrointestinal tract could ameliorate IBD by promoting the colon epithelium repair [90] and improving the glucagon-like peptide-2 production [91]. In the previous study, GPR43 has been found in the neutrophils and colon [92]. GPR43 is vital to neutrophil recruitment in intestinal inflammation and their deficiency induces severe inflammation. SCFAs would bind to GPR41/43 and influence anti-inflammation activity in the cells [85]. SCFAs metabolized from natural polysaccharides also stimulate the GPR43 on colonic T cells followed by up-regulating the expression of Foxp3 [90] and facilitating peripheral T-reg cells, which finally inhibits the inflammatory response in the intestinal mucosa [35]. Otherwise, activated GPR43 involves the NF-κB pathway, which inhibits the expression of pro-inflammatory cytokines such as IL-6 and IL-1β. It also indicates that GPR43 is beneficial to attenuate the inflammation responses in the gut [93]. β-arrestin1, one of the important regulators of the GPR signaling pathway [94], makes a crucial contribution in the activation of nucleotide leucine repeat pyrin 3 (NLRP3) inflammasome [95]. NLRP3 inflammasome exerts importance in the inflammation and immunity responses via the caspase-1 activation and IL-1β maturation. The motivation of NLRP3 inflammasome could aggravate gut inflammation [96] and the variant of NLRP3 in the cells would trigger severe IBD [86]. Therefore, the pro-inflammatory effects of β-arrestin1 and NLRP3 were mediated through the cytokines in the inflammatory intestine like the DSS-induced colitis models. However, the researchers have offered evidence that administration of *Dendrobium officinaleon* polysaccharides (DOPS) ameliorated the injury of the colon and moderated the symptoms of colitis including the rapid body weight loss and the somatotype decreased in DSS-induced mice. The inhibition effect of DOPS to the NLRP3 inflammasome pathway was showed by down-regulation of the IL-1β, IL-18, and caspase-1 expression. The results also displayed that DOPS could suppress the β-arrestin1 signaling pathway. Moreover, in vitro experiments indicated that DOPS significantly inhibit their expressions in the LPS-stimulated NCM 460 cells, the results were the same as before in vivo [97]. Above all the results have indicated that natural polysaccharides could regulate the expression of GPRs on behalf of alleviating the IBD.

### 3.3. Increase Intestinal Integrity via Up-regulating Tight Junction Proteins

**Figure 2:**
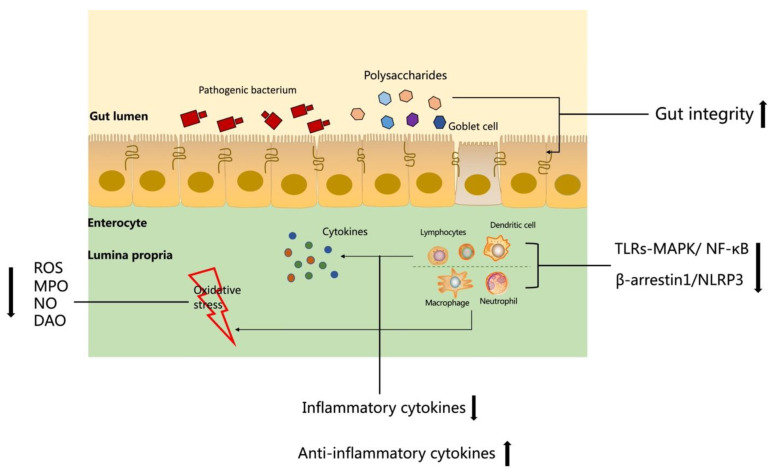
The summary of mechanisms about polysaccharides involved in IBD patient intestine.

Gut integrity is vital to maintaining the host’s innate immunity, which could prevent intestine damage from lipopolysaccharides (LPS) and toxins [98]. The tight junction proteins such as the ZO family, claudins, and occludins are responsible for the diffusion of epithelium and among cells. Numerous inflammatory responses would affect the tight junction proteins expressions and since colitis makes the levels change, the expression of tight junction proteins is always decreased in IBD [99]. TNF-α plays an important role in the mediation of occludin internalization, and the occludin has a positive relationship with gut permeability. Although the cytokines increase the gut permeability, overexpression of occludin could ameliorate it [100]. Moreover, the other mechanism involved in gut integrity regulation is TNF-α activation of the NF-κB signal transduction pathway. The inhibition of NF-κB could prevent colitis mice from having diarrhea and serious loss of water, which demonstrated the modulatory function of NF-κB in barrier capability [101]. In the colitis patient, TNF-α and IL-1β not only increase the infiltration of neutrophils, but also cause damage to the intestinal barrier, which may induce diarrhea symptoms in patients [102]. Otherwise, TNF-α attracts the myosin light-chain phosphorylation, which damages the tight junction protein. Then the risky factors and microbes like LPS, the signals from gram-negative bacteria, could enter the intestine through the mucous membrane leak, which would bind to toll-like receptors and stimulate the TLR-mediated signaling pathway [103], further provoking the inflammatory response. IFN-γ diminishes the ZO-1 and occluding expression through the adenosine monophosphate-activated protein kinase dependent pathway without regard to the energy level of the cell. Therefore, it also could increase barrier permeability [104]. FCPS treatment could ameliorate colitis symptoms by restoring the expression of light junction protein claudin-1 and down-regulating the level of TNF-α and IL-1β in the DSS-induced colitis mice [82]. The animal models’ results proved that FCPS could facilitate the repair of damaged intestinal barriers, thus blocking the penetration of endangering molecules and microbes through the mucous membrane [105]. In addition, the gastrointestinal protective effect of DIP has been reported to recover the expression level of claudin-1, occludin, and ZO-1 in the DSS-induced colitis mice [27]. These studies indicated that despite the fact that these inflammatory cytokines are always presented in the intestine at the same time during inflammation and harmful to the patient due to the disconnection of the tight junction proteins [106], the natural polysaccharides could restrain the response of colon inflammation and promote IBD recovery via increasing the tight junction protein expression in the gut and thereby protect the colon structure of the IBD patient (Figure 2).

### 3.4. Regulation of Oxidative Stress

In the intestine, immunity plays a critical role in the pathogenesis of IBD. As the inflammation induces the T-cell response through the secretion of inflammatory cytokines, the other activated immune cells, including macrophages and neutrophils [107], induce significantly increased production of oxygen free radicals, which cause further damage in colon tissue. The synthesis of oxygen free radicals and the antioxidase in the intestine, including ROS, myeloperoxidase (MPO), nitric oxide (NO), malondialdehyde (MDA), plasmic diamine oxidase (DAO), and glutathione (GSH). ROS have various physiological functions but under the condition of oxidative stress, overproduction of it is harmful to intestine cell membrane lipids [108], DNA, and proteins. ROS also are responsible for causing diarrhea, one of the most common symptoms in IBD, due to the excessive secretion of water and electrolytes [109]. MPO, as one of the reactive oxygen species, is always regarded as the inflammatory marker of neutrophil infiltration. However, polysaccharides might ameliorate damage through inhibiting neutrophil infiltration in the gut. Han et al. have treated DSS-induced mice with SP and found the expression level of MPO significantly decreased in the gut [44].

After being stimulated by oxidative stress, iNOS belonging to reactive oxygen/nitrogen species (ROS/RNS)-generating systems catalyzes the production of NO. As one of the markers of inflammation, NO is released by the neutrophils in damaged tissues and induces an immunity inflammatory response, whose generation has a positive relationship with the level of oxygen free radicals [26]. Prior research has shown that administrated FVP would lead to a decline in NO content in the gut of DSS-induced colitis models [34]. Except for promoting the production of NO, iNOS also improves the expression of COX-2 in the meanwhile. COX-2 could catalyze prostaglandin production and finally induce the inflammation response. Shao et al. administrated acetic acid-induced mice with EP-1 and found both the expression of COX-2 and related mRNA were down-regulated, which might prove that polysaccharides could balance the immunity in the intestinal system via regulating the oxidative stress, following subsequently moderate IBD [14]. The above results have indicated that polysaccharides make a critical contribution to the down-regulation of reactive oxygen species and therefore exert an essentially protective effect on the colon of patients.

The different concentration of MDA and GSH in the tissues has revealed that diverse levels of oxidants caused by the epithelial cell rupture. Dutra et al. have confirmed that *G. caudata* PLS suppresses the acetic acid-induced UC mice [43], which induces the restoration of the GSH and down-regulation of MDA level in the cells. In the pathogenesis of IBD, above all the results have proved that polysaccharides could decrease the production of reactive oxygen species and their metabolites as well as increase the level of anti-oxidant molecules meanwhile to ameliorate IBD that is caused by prooxidant substances.

## 4. Future Outlooks and Conclusions

Natural polysaccharides have the potential ability to be used in IBD treatment. Many types of polysaccharides from mushroom, seaweed, herbs, and plants can be fermented in the colon, which not only changes the diversity of gut microbiota, but also recovers gut health via stimulating various types of immune cells and motivating numerous immunity-related signaling pathways. The main mechanism of polysaccharides on inflammatory bowel disease relies on immune regulation, anti-oxidation, and regulation of probiotics in the intestine.

Natural polysaccharides have attracted considerable attention because of their low side effects, nontoxicity to individuals, and easy availability in diet. However, further examination is necessary to explore the link between natural polysaccharides and immune regulation in diseases, together with analyzing the variety of microbiota in the intestine. Systemic studies would help create better understanding of the specific mechanism that the function of polysaccharides exert on diseases. Thus, complementary alternative treatments could be provided to IBD patients. Additionally, for the purpose of better applying polysaccharides to normal immunity recovery, more clinical work is needed on the related foods and drugs to ensure the safety of dosage and half-life time. More innovative polysaccharide-based foods and medicines for IBD can be expected under a comprehensive understanding of the structure, biological activities, and underlying mechanism of polysaccharides.

## Figures and Tables

**Table 1:** Summary of the effects of some natural polysaccharides on inflammatory bowel disease amelioration.

Name	Source	Origin	Molecular Weight (kDa)	Model	Targets	Reference
EP-1	*Hericium erinaceus*	Mushroom	3.1	Acetic acid-induced mice	Recover the level of *Firmicutes, Bacteroidetes, Proteobacteria,* and *Actinobacteria*	[17]
HECP	*Hericium erinaceus*	Mushroom	87	DSS-induced colitis mice	Decrease expression of pro-inflammatory cytokines (IL-6, IL-1β, TNF-α), COX-2, iNOS	[18]
HCP	*Sarcodon aspratus*	Mushroom	670	RAW264.7 cells	Increase the expression of NO, NOS, cytokines and phagocytic activity Stimulate TLR4-MAPK-NFkB signaling pathway	[21]
DP1	*Dictyophora indusiata*	Mushroom	1132	RAW264.7 cells	Increase the level of *Bacteroidaceae* and *Enterobacteriaceae* Reduce the pro-inflammatory cytokines (TNF-α, IL-1β, IL-6, IFN-γ), MPO, NO and enhance anti-inflammatory cytokines (IL-4, IL-10), T-SOD; Inhibit CD86 Increase tight junction proteins including claudin-1, occludin, and ZO-1; and expression of Bcl-2 and Bax	[22,23,24]
FVP	*Flammuliana velutipes*	Mushroom	dozens to hundreds	DSS-induced colitis mice	Decrease the level of MPO, NO, and increase SOD Raise the ratio of *Firmicutes/Bacteroidetes* and enhance the abundance of *Lachnospiraceae*	[34]
PEP	*Pleurotus eryngii*	[34]	426	C57BL/6 mice	Increase the abundance of *Porphyromonadaceae, Rikenellaceae, Bacteroidaceae,* and *Lactobacillaceae* Decrease SCFA production	[41]
EPA-1	*Pleurotus eryngii*	Mushroom	99.7	RAW 264.7 cells	Increase the production of pro-inflammatory cytokines TNF-α, IL-1, and IL-6 Increase expression level of phosphorylated p38, ERK, JNK, and NF-κB	[43]
SP	*Graciliaria lemaneiformis*	Seaweed		DSS-induced colitis mice	Inhibit activation of MPO Repress intestinal endotoxin and lipopolysaccharide-binding protein production Decrease the expression of TNF-α, IL-6, IL-1β	[44]
BMP	*Blidingia minima*	Seaweed		DSS-induced mice	Decrease the expression of MPO and EPO Increase the expression of IL-10 and decrease the production L-1β and TNF-α, NF-κB, IκB-α, and AKT	[46]
ALP-1	*Arctium* *lappa* L.	Chinese herbs	5.12	Systemic inflammatory mice	Decrease the IL-1β, IL-6, TNF-α; increase IL-10 and Ig A; Increase the abundance of *Lactobacillaceae, Lachnospiraceae,* and *Ruminococcaceae* and inhibit the abundance of *Bacteroides* and *Staphylococcus*	[45,46]
ASPP	*Arctium* *lappa L.*	Chinese herbs	120	LPS-induced inflammatory cell	Increase the expression of IL-10 and decrease MPO Increase the abundance of *Firmicutes, Alistipes, Odoribacter,* and the *Firmicutes/Bacteroides* ratio	[52]
NFP	*Morinda* *citrifolia*	Chinese herbs	456	DSS-induced mice	Improve the tight junction proteins production including zonula, occludens-1, and occludins	[60]
APS	*Astragalus membranaceus*	Chinese herbs		DSS-induced mice	Down-regulating production of MPO, NF-κB, and pro-inflammatory cytokines (TNF-α, IL-1β, IL-6)	[62]
DOP/EDOP	*Dendrobium officinale*	Chinese herbs		DSS-induced mice	Inhibit NLRP3 inflammasome and β-arrestin1 Decrease the expression of pro-inflammatory cytokines including IL-1β, IL-6, and TNF-α; Activate GPR41/43 signaling pathway Increase the abundance of *Bacteroides, Lactobacillus* plus *Ruminococcaceae*, but down-regulate the levels of *Proteobacteria* and *Akkermansia*	[64]
LBP	*Goji berry*	Chinese herbs	2.1−6.5 × 10^3^		Inhibit the expression of CXCL1, MCP-1, COX-2, and IL-6 Increase the abundance of *Akkermansia, Lactobacillus,* and *Prevotellaceae* Increase the expression level of TGF-β and sIgA	[70]
CREP	*Codonopssi pilosula*	Chinese herbs	2.0 × 10^3^/7.3	DSS-induced mice	Down-regulation of TJ proteins including Occludin, ZO-1, claudins, and MUC-2 Up-regulation of IL-22	[73]
WPSPP-1	*Purple sweet potato*	Plant	10^3^	DSS-induced mice	Increase the expression of pro-inflammatory cytokines IL-10 and decrease the expression of IL-1β, TNF-α, IL-6. Promote the level of SOD and T-AOC, and inhibit the production of MDA	[54]
ASPP	*Purple sweet potato*	Plant		DSS-induced mice	Inhibit the pro-inflammatory cytokines including IL-1β, IL-6, and TNF-α Increase the production of SCFAs	[78]
TFPS	*Camellia sinensis* L.	Plant	4.4/31	Human stool samples	Increase the abundance of *Escherichia/Shigella, Enterococcus, Collinsella, Lactobacillus,* and *Bifidobacterium* Decrease the abundance of *Enterobacter, Streptococcus, Bacteroides, Clostridium XlVa, Megasphaera, Roseburia, Granulicatella, Akkermansia,* and *Fusobacterium*	[81]
FCPS	*Ficus carica*	Plant	98.9	DSS-treated mice	Decrease expression of IL-1β, iNOS, TNF-α, MCP1, IL-6, and COX-2 Decrease the abundance of *Esherichia* and *Clostridium* Increase the abundance of *Prevotella, Bacteroides, Butyricicoccus,* and *Coprococcus*	[83]

**Table 2:** The mechanisms of natural polysaccharides involved in colon inflammation amelioration.

Mechanism Involved	Source of Polysaccharides	Results	Reference
MAPK transduction signaling pathway	*Ganoderma lucidum and G. sinense/Pleurotus eryngii*	Increase the phosphorylation level of ERK, JNK, and p38 in macrophage cells	[38,84]
	*Dictyophora indusiata*	Decrease the phosphorylation level of ERK and pro-inflammatory cytokines in DSS-induced mice	[27]
NF-κB signaling pathway	*Blidingia minima/H. erinaceus/Arctium lappa L./Purple sweet potato*	Restore phosphorylation level of NF-κB, IκB-α, and AKT in colitis mice model	[18,46,53]
G-protein-coupled receptors	*Hericium Erinaceus*	Increase the expression of GPR41/43 in acetic acid-induced colitis mice	[85]
	*Dendrobium officinaleon*	Inhibit β-arrestin1and NLRP3 inflammasome signaling pathway in DSS-induced mice	[86]
Increase intestinal integrity via upregulate tight junction proteins	*Ficus carica*	Increase the expression of light junction protein claudin-1 and decrease the expression of TNF-α and IL-1β in DSS-induced mice	[82]
	*Dictyophora indusiata*	Increase the expression of claudin-1, occludin, and ZO-1 in DSS-induced mice	[27]
Regulation of oxidative stress	*Graciliaria lemaneiformis/Flammuliana velutipes*	Decrease the expression of MPO and NO in DSS-induced mice	[34,44]
	*Hericium Erinaceus*	Decrease the expression of COX-2 in acetic acid-induced mice	[14]
	*Graciliaria caudata*	Restoration of GSH and decrease of MDA in acetic acid-induced mice	[43]

## Data Availability

Data is contained within the article.
